# Screening of Pesticides with the Potential of Inducing DSB and Successive Recombinational Repair

**DOI:** 10.1155/2017/3574840

**Published:** 2017-10-10

**Authors:** Karen Suárez-Larios, Ana-María Salazar-Martínez, Regina Montero-Montoya

**Affiliations:** Departamento de Medicina Genómica y Toxicología Ambiental, Instituto de Investigaciones Biomédicas, Universidad Nacional Autónoma de México, Apartado Postal 70228, 04510 Ciudad de México, Mexico

## Abstract

A study was realized to ascertain whether eight selected pesticides would induce double strand breaks (DSB) in lymphocyte cultures and whether this damage would induce greater levels of proteins Rad51 participating in homologous recombination or of p-Ku80 participating in nonhomologous end joining. Only five pesticides were found to induce DSB of which only glyphosate and paraoxon induced a significant increase of p-Ku80 protein, indicating that nonhomologous end joining recombinational DNA repair system would be activated. The type of gamma-H2AX foci observed was comparable to that induced by etoposide at similar concentrations. These results are of importance since these effects occurred at low concentrations in the micromolar range, in acute treatments to the cells. Effects over longer exposures in actual environmental settings are expected to produce cumulative damage if repeated events of recombination take place over time.

## 1. Introduction

Pesticides constitute a heterogeneous group of chemicals, specifically synthesized to control plagues, weeds, and unwanted organisms of all kinds [1]. In 2016, the United States Environmental Protection Agency [2] had about 1,140 active ingredients registered, both organic or inorganic [3]. The authorized organic pesticides include several chemical groups or families [4]. They started being used by the year 1948 in Mexico with DDT and other organochlorides, followed by organophosphates, carbamates, pyrethroids, and herbicides like paraquat and 2,4-D [5].

These compounds are of wide spectrum and show varying degrees of toxicity, not only for target species, but to others, unintendedly, including humans [6]. In agriculture, pesticides are typically applied in mixtures and exposure to these compounds has been associated with chronic adverse health effects including neurological (cognitive problems and Parkinson disease), reproductive, respiratory (asthma), metabolic (diabetes and obesity), and developmental problems and cancer [3, 6], particularly infant and childhood leukemia [7–9] in relation to which they have been described as promoters [8, 10] since many of them do not induce genetic damage. Genotoxic effects, however, have been documented in ecotoxicological [11] and epidemiological studies [12–16], in animal models [17–20], as well as* in vitro* [21–32]. Biomarkers identified include sister chromatid exchanges, chromosomal aberrations, micronuclei, and DNA breaks observed in the comet assay. Furthermore, there are studies suggesting that pesticides produce translocations associated with childhood leukemia [33–35]. So, it is possible that some of these compounds may induce reciprocal translocations which have been identified in relation to certain subtypes of leukemia, such as t(4;11), t(8;21), and t(12;21) [10, 36].

These alterations can originate in double strand breaks (DSB) and the principal repair mechanisms for these lesions are the canonical nonhomologous end joining (c-NHEJ) and homologous recombination (HR) [37, 38]. It is known that ionizing radiation, benzene, and antineoplastic chemicals, identified as leukemogenic, induce this kind of damage [39–42]. As pesticides have been related to leukemia [8, 34], we wanted to ascertain whether they would induce these lesions that are the primary event in the formation of chromosomal translocations like the ones described [37].

The goal of the present study is to determine whether selected pesticides are capable of inducing DSB in an* in vitro* model and the recombinational pathway ensuing this damage.

## 2. Material and Methods

### 2.1. Donors

Healthy young male donors were asked to participate in the study which was explained to them and then were asked to donate 3 ml of blood. They were 21 to 35 years old, nonsmokers, and not alcoholics. Previously to donating the blood, they had not taken medication or were not subjected to radiation for medical purposes. Blood samples were taken with heparinized syringes (Monovette, Sarstedt).

### 2.2. Reagents

Pesticides endosulfan, glyphosate, pentachlorophenol, permethrin, propoxur, and paraoxon and the metabolites AMPA (aminomethylphosphonic acid, from glyphosate) and endosulfan lactone (from endosulfan) as well as etoposide were purchased to Sigma-Aldrich, Mexico (Table 1). DMSO was purchased from ATCC. The CellTiter 96 AQueous One Solution Cell Proliferation reagent from PROMEGA was used to determine cytotoxicity. The high performance chemiluminescence film kit and the Amersham HyperFilm^TM^ ECL film from GE Healthcare were used for the protein analysis.

### 2.3. Antibodies

Primary antibodies used were mouse anti-phospho-histone H2AX (Ser 139) and rabbit anti-Rad51 polyclonal antibody, purchased from Millipore; rabbit anti-Ku80 (phosphoT714) polyclonal antibody from Abcam; goat anti-Actin (1-19) polyclonal antibody from Santa Cruz. Secondary antibodies used were Alexa-Fluor 555 goat anti-mouse from Invitrogen, goat anti-rabbit IgG-HRP, goat anti-mouse IgG-HRP, and donkey anti-goat IgG-HRP from Santa Cruz. Mounting medium with DAPI was purchased from Vectashield.

### 2.4. Treatments to Evaluate Double Strand Breaks (DSB)

Four serial dilutions of each pesticide or metabolite, as well for etoposide, were tested (Table 1). Treatments were done in duplicate as follows: 250 microliters of whole blood were placed in 2.25 ml of RPMI-1640 and treated with the corresponding compound and concentration for 1.5 h at 37°C, after which 3 ml of 0.075 M KCl was added and incubation was continued for 30 min. Lymphocytes were then fixed according to the protocol by Andrievski and Wilkins [43] with minor modifications; briefly, cells were recovered by centrifugation at 250*g* for 10 min at room temperature; the supernatant was removed and formaldehyde was added at a final concentration of 4%. Ten min later, 1 ml PBS with 0.12% Triton X-100 was added, and an incubation of 30 min was allowed at room temperature; thereafter the samples were washed with 1 ml cold PBS supplemented with 4% fetal bovine serum (FBS) and centrifuged for 8 min at 300*g* at 0°C. Supernatant was discarded and 1 ml of cold 50% methanol in PBS was added. The samples were left at −20°C all night. Tubes were then centrifuged at 300*g* for 8 min at 0°C, the supernatant was discarded, 3 ml of cold methanol was added, and samples were kept at −20°C until analysis.

### 2.5. DSB Identification

Phosphorylated histone H2AX foci were detected by immunofluorescence under the microscope (Nikon Eclipse 80i). Staining of lymphocyte nuclei was done according to Watters et al. [44] with minor modifications. Slides were washed three times with PBS during 30 min and blocked with KCMT buffer with 12% FBS (120 mM KCl, 20 mM NaCl, 10 mM Tris-HCl pH 8, 1 mM EDTA, and 0.2% Triton X-100) for 1 h at room temperature. Primary anti-phospho-histone H2AX (Ser139) diluted 1 : 200 in blocking solution was added and was left to incubate overnight at 4°C. Three washes with KCMT were done, 15 min each. Incubation with the secondary antibody was done: Alexa-Fluor 555 goat anti-mouse diluted 1 : 500 (in blocking solution) for 1 h at room temperature. The slides were washed as with the primary antibody and were rinsed in deionized water before mounting in DAPI mounting medium. They were analyzed for gamma-H2AX foci under a fluorescence microscope with the adequate filters.

Evaluation of foci was done in 2 slides per concentration. 50 cells were evaluated in three different regions per slide and foci were counted on them; in total 300 cells were evaluated per treatment. When a nucleus presented 1 or more foci, it was considered positive, according to Scarpato et al. [45]. The extent of DNA damage was classified in three categories: percentage of undamaged cells (without gamma-H2AX foci), with moderated damage (<10 gamma-H2AX foci) and with severe damage (>10 gamma-H2AX foci). Additionally, the damage was expressed as mean percentage of gamma-H2AX positive nuclei.

### 2.6. Cytotoxicity

Cytotoxicity was tested using The CellTiter 96® AQueous One Solution Reagent form PROMEGA and following the general protocol suggested by the manufacturer. Mononuclear cells were isolated from blood using Histopaque-1077 (Sigma). Cells were plated in 96-well plates, 100,000 cells per well, and treated with the compounds, each concentration in triplicate. Treatment took place during 1.5 h at 37°C, after which the reagent was added and incubated for 3 h at 37°C and absorbance at 490 nm was recorded using a 96-well plate reader (Synergy H4 Hybrid, Biotek). The percentage of survival for each treatment was calculated using the following formula: (Abs490 nm of treatment/Abs490 nm of negative control) × 100.

### 2.7. Western Blot Analysis of Proteins Participating in DNA Recombination

This analysis was done only with compounds that induced gamma-H2AX foci (Table 1). Three serial concentrations were tested in duplicate. Mononuclear cells were isolated with Histopaque-1077. Treatments were applied to cells resuspended in 1 ml RPMI-1640 (500,000 cells per tube) during 1.5 h at 37°C, after which cells were centrifuged at 3000*g* for 5 min at 4°C. The supernatant was discarded and 600 microliters of 10% 0.5 M sodium azide in PBS was added and vortexed. Centrifugation was done again, the supernatant was discarded, and the cell pellets were kept at −70°C until used. Two separated experiments were made per compound, with two donors each time.

#### 2.7.1. Protein Extraction and Quantification

RIPA lysis solution containing phosphatase and protease inhibitors was added to each cell pellet. Samples were then sonicated with one pulse, incubated in ice for 15 min, and centrifuged at 13,800*g* for 15 min. The supernatant was recovered in a 0.5 ml tube. Five microliters of each sample was placed in a 96-well plate for protein quantification. The Lowry assay was performed with the DC Protein Assay kit (Bio-Rad); the plate was agitated in the dark for 15 min at room temperature. The concentration was then determined in a plate reader (VersaMax Tunable) at 750 nm. The samples were then stored at −70°C until use.

#### 2.7.2. Electrophoresis and Transfer

Phosphorylated Ku80 (phospho-T714) and Rad51 were the proteins evaluated. Beta-actin was used as internal control. 35 micrograms of total protein was separated in a 10% SDS-polyacrylamide gel and transferred to a nitrocellulose membrane (0.45 micrometers, GE-Healthcare) with a Trans-Blot® SD Semi-Dry Transfer Cell (Bio-Rad). The membrane was incubated with blocking solution (2% blotting-grade blocker milk in TBS) at 4°C overnight and gentle agitation. Two washes were then made with TBS-1% Tween, 10 min each, and one with TBS for 5 min. The membrane was cut at the appropriate level and incubation with each primary antibody was separately set at 4°C overnight with gentle agitation: rabbit anti-Rad51 (1 : 500 in blocking solution) or rabbit anti-phosphorylated Ku80 (p-Ku80) (1 : 1000 in blocking solution). Incubation with primary antibody goat anti-actin (1 : 1000 in blocking solution) was done at 28°C for 1 hr. Membranes were then washed with TBS-1% Tween three times, 10 min each, and with TBS for 5 min at room temperature. Incubation with the secondary antibody was done: goat anti-rabbit IgG-HRP 1 : 3000 (in blocking solution) for the first two antibodies and donkey anti-goat IgG-HRP 1 : 3000 (in blocking solution) for the latter, for 1 h at 28°C and agitation. Rinsing was made as with the primary antibodies, and the membranes were exposed and revealed with a luminescence kit (GE Healthcare). Acquisition of optical densities was done with Quantity One software (Bio-Rad), version 4.1.1. Values obtained for each protein (Rad51 or p-Ku80) were normalized with respect to beta-actin and the mean of normalized negative controls; results are presented as the % with respect to normalized negative controls [46]. Two membranes were done per separated experiment with each compound tested; that is, four data items were obtained for each concentration of the compounds analyzed.

### 2.8. Statistical Analyses

Statistical calculations were realized with the GraphPad Prism 6 software package: results for gamma-H2AX foci and optical density from western blot were evaluated with the Kruskal-Wallis test and Dunn's multiple comparison as a post hoc test; the value of etoposide as a positive control at the concentration of 50 microM was analyzed with Mann–Whitney *U* test with respect to the negative control, establishing a *p* value for significance of <0.05 for all tests. Cytotoxic effect of treatments was analyzed with linear regressions, establishing a *p* value for significance of <0.05.

## 3. Results

### 3.1. DSB Identification

Among the compounds tested, four showed a significant effect on the number of cells with DSB (Table 2), whereas endosulfan, propoxur, and AMPA showed no effect on this parameter. Pentachlorophenol had a hormetic behavior, inducing gamma-H2AX foci in the lowest concentrations, whereas the effect diminished in the highest (Table 2). As explained in Material and Methods, three categories of nuclei with foci were enumerated: (1) without gamma-H2AX foci, (2) with one to ten foci, and (3) with more than ten foci or those who had clustered foci which could not be enumerated. Endosulfan lactone, permethrin, pentachlorophenol, glyphosate, and paraoxon mainly produced cells showing 1 to 10 foci which were not in relation to the concentration but showed a similar increase in every concentration tested, paraoxon being the only one which showed a consistent increase of DSB with increasing concentrations (*R*^2^ = 0.1236, *p* = 0.0321) (Table 2). However, glyphosate and paraoxon showed an increase of cells with more than 10 foci (Table 2), related to the concentration (linear regression,* R*^2^ = 0.2, *p* = 0.02 for glyphosate;* R*^2^ = 0.35, *p* = 0.0003 for paraoxon). Results for lower etoposide concentrations tested (0.4, 2, and 10 microM) are not presented in this table but in the figure presented in Discussion; a significant correlation with the dose was found,* R*^2^ = 0.82 and *p* < 0.0001 for cells with more than 10 foci.

### 3.2. Cytotoxicity

The same concentrations used to determine DSB were used to assess a cytotoxic effect of the five compounds that produced DSB, finding that glyphosate and endosulfan lactone reduced the number of viable cells in a dose-dependent manner (Table 3), going from 100% viability to 70% with glyphosate and from 100% to 60%, with endosulfan lactone. Pentachlorophenol, permethrin, and paraoxon did not show a cytotoxic effect at the concentrations used.

### 3.3. Quantification of p-Ku80 and Rad51 Proteins

**Figure 1:**
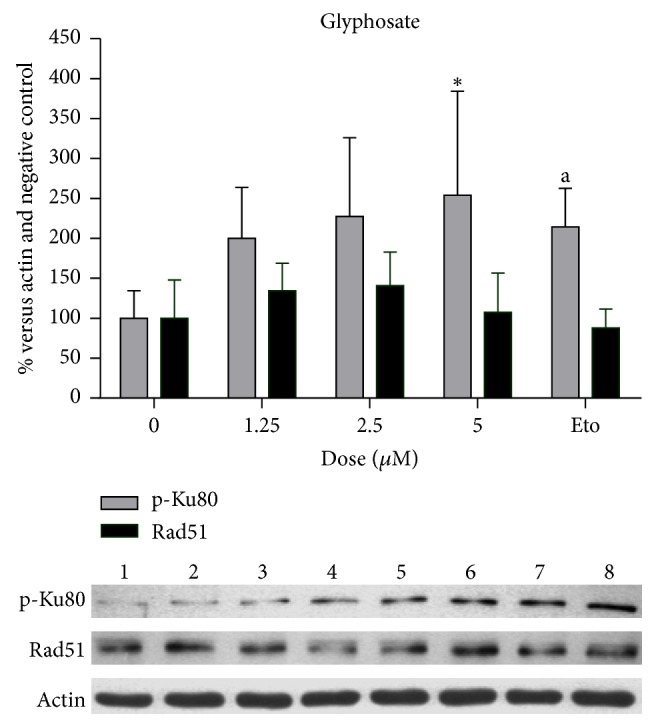
Graph: p-Ku80 protein increased due to treatments with glyphosate in a dose-dependent manner (linear regression, *p* < 0.05). Individual concentrations were analyzed by Kruskal-Wallis and Dunn's multiple comparison test; the asterisk shows that 5 microM was significantly different from the negative control. The bottom part shows a representative membrane: lane 1, negative control; lanes 2–4, pentachlorophenol (0.2, 1.0, and 5 microM); lanes 5–7, glyphosate (1.25, 2.5, and 5 microM, see graph); lane 8, etoposide (10 microM).

**Figure 2:**
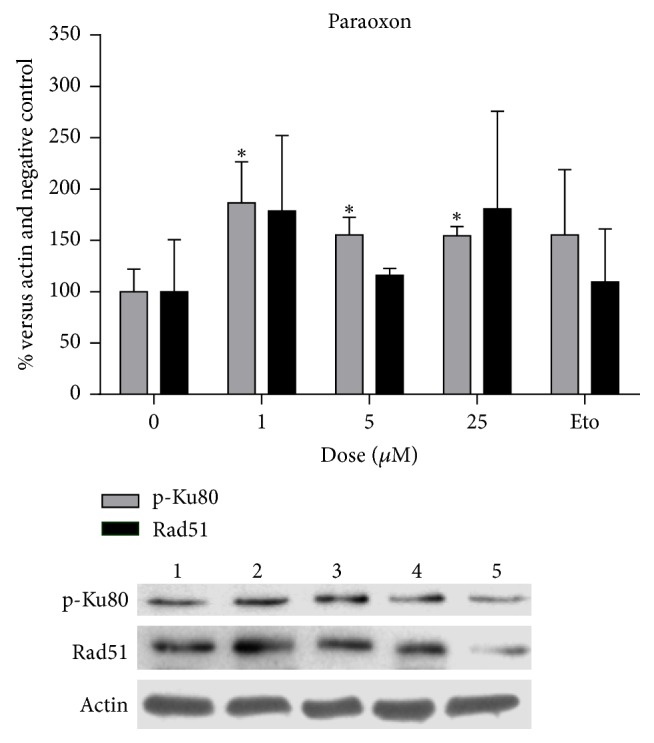
Increase in the amount of p-Ku80 protein in cells treated with paraoxon: lane 1, negative control; lanes 2–4, paraoxon (1, 5, and 25 microM); lane 5, etoposide (10 microM). Rad51 did not show significant variation among treatments as is shown in the graph.  ^*∗*^Significantly different dose; Kruskal-Wallis; Dunn's post hoc test, *p* < 0.05.

The five compounds positive to DSB production were used in a set of treatments to determine whether DNA recombination would be induced; pentachlorophenol, even though it induced DSB only in the lowest concentrations tested, was also included in these analyses. P-Ku80 was used to evaluate NHEJ and Rad51, to evaluate HR (Table 4). Glyphosate was found to significantly induce the presence of p-Ku80 in a dose-dependent manner (*p* < 0.05, Figure 1), whereas Rad51 was not significantly affected. Paraoxon induced p-Ku80 as well, however, not in a dose-dependent manner (Figure 2). The rest of the compounds tested did not show an effect on neither of the proteins studied. Etoposide, the positive control, consistently induced p-Ku80, although with a wide variation between tests, at the concentration used for this analysis (10 microM) (Figures 1 and 2).

## 4. Discussion

A search was done to evaluate whether eight known pesticides still in use in our country could induce DSB, a lesion related to the formation of chromosomal rearrangements and leukemia risk.

Four of the compounds tested exhibited an ability to induce this kind of DNA damage, recognized in the form of phosphorylated H2AX foci in the nuclei of normal human lymphocytes, in at least two of the concentrations tested.

**Figure 3:**
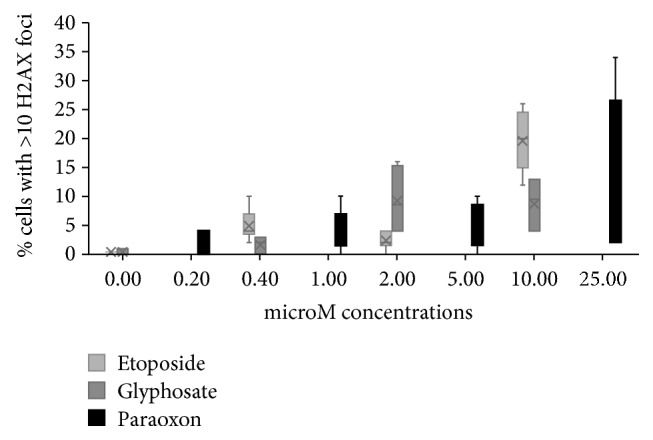
Percentage of cells with more than 10 gamma-H2AX foci induced by treatments with etoposide, glyphosate, and paraoxon. The three compounds induced an increase related to the dose; see Results for statistical values of linear regression analyses.

It is noticeable that etoposide, an antineoplastic agent, at the concentration used as a positive control of 50 *μ*M, induced more than 85% of cells with more than 10 foci; however, in lower concentrations etoposide induced foci in a comparable manner as was observed with glyphosate and paraoxon (Figure 3). This amount of damage seems to be of relevance in relation to the increase of repair proteins like p-Ku80, since only glyphosate and paraoxon produced a significant increase in this protein, whereas the rest of the compounds that induced lower amounts of gamma-H2AX foci did not. In this set of experiments to evaluate proteins participating in DNA recombination, treatments with glyphosate were modified to avoid cytotoxicity, 5 microM being the highest concentration tested. It should be said that our results differ from those of Townsend et al. [47] who found cytotoxicity caused by glyphosate in Raji cells only at higher concentrations (10 mM); this difference indicates that normal human cells are more sensitive to the toxic effects of the compound. Etoposide concentration as a positive control was also lowered to 10 microM to make it more comparable with the treatments with pesticides. As expected, Rad51 was not induced by the treatments given to nonproliferating lymphocytes. This is consistent with previous reports indicating that the HR repair mechanism does not participate in DSB repair in cells in G0/G1 [48–52].

The results presented here point to paraoxon and glyphosate, an insecticide and a herbicide both organophosphates, as inducers of DSB in human cells. Paraoxon is a metabolite of parathion, a compound classified by the WHO toxicity classification [53] as Ia, extremely hazardous, and both are cholinesterase inhibitors, whereas glyphosate toxicity is under discussion in many fora. The treatments given to the cells in a nonproliferative state induced not only the breakage of DNA, but also the phosphorylation of Ku80, a protein that participates in the c-NHEJ repair pathway. These results agree with those reported in studies with peripheral blood mononuclear cells with DSB induction by radiation, Frasca et al. [54] found that Ku80 was phosphorylated prior to the formation of the Ku70/Ku80 dimer required for the initiation of repair, whereas Shelke and Das [55] detected an upregulation of Ku80 and other proteins that participate in the c-NHEJ repair mechanism. This c-NHEJ repair pathway is known for being prone to error, introducing microdeletions or microinsertions which could be mutagenic and alter cell behavior if they occur in coding or regulatory sequences [56]. This is one possible outcome of the DSB in nonproliferating cells. Another possibility is that the damage observed was extensive enough as to induce the intervention of the alternative NHEJ (a-NHEJ), via the phosphorylation of Plk-3 and CtIP, necessary for the activation of a-NHEJ in G0/G1 as demonstrated by Barton et al. [57]. This pathway has been demonstrated to be the only one responsible for the formation of chromosomal translocations, of great concern in the development of leukemia, lymphoma, and secondary cancers [51, 56]. Etoposide, our positive control, is well known as a topo II inhibitor capable of producing complex DSB (defined as many DSB in close proximity, [57] in G0, acting in a similar way as ionizing radiation and producing chromosomal rearrangements in all phases of the cell cycle [40, 58]; the damage induced by this compound in this study was comparable to the damage induced by paraoxon and glyphosate (DSB and Ku80 induction), so the question emerging from these results is whether the outcome for the cells damaged by paraoxon and glyphosate would be similar to the outcome of cells damaged by etoposide and they would also induce chromosomal rearrangements.

## 5. Conclusions

Eight pesticides were tested for their ability to produce DSB in nonproliferating lymphocytes and to evaluate whether the classical recombinational mechanisms of DNA repair would come into action. Two of them, paraoxon and glyphosate, were found to produce both DSB and the phosphorylation of Ku80, a protein participating in the c-NHEJ recombinational repair pathway.

These results are of importance since these effects occurred at low concentrations in an acute treatment to the cells. Effects over longer exposures in actual environmental settings are expected to produce cumulative damage if repeated events of recombination take place over time.

## Figures and Tables

**Table 1:** Treatments applied for DSB and protein studies.

Compound	Concentrations (*µ*M)		CAS number
	DNA damage	Protein studies	
Endosulfan	0, 0.01, 0.08, 0.4, 2	—	115-29-7
Glyphosate^*∗*^	0, 0.4, 2, 10, 50	1.25, 2.5, 5	1071-83-6
Pentachlorophenol^*∗*^	0, 0.03, 0.15, 0.75, 3.75	0.2, 1, 5	87-86-5
Permethrin^*∗*^	0, 8, 40, 200, 1000	8, 40, 200	52645-53-1
Propoxur	0, 0.24, 0.48, 0.96, 1.44	—	114-26-1
AMPA	0, 40, 200, 1000, 5000	—	1066-51-9
Endosulfan lactone^*∗*^	0, 0.08, 0.4, 2, 10	0.017, 0.05, 0.15	3868-61-9
Paraoxon^*∗*^	0, 0.2, 1, 5, 25	1, 5, 25	311-45-5

Etoposide^*∗*^	0, 0.4, 2, 10, 50^3^	10, positive control	33419-42-0

^*∗*^These compounds were used for protein studies;  ^3^50 microM was used as positive control with each pesticide.

**Table 2:** Percentage of cells with *γ*-H2AX foci due to treatments.

Compound	Doses (microM)	Mean of % cells (S.D)				Median of % cells with total damage	*p* ^*∗*^ * (chi-squared value)*
		Without foci	With 1 to 10 foci	With more than 10 foci	With total damaged		
Glyphosate	0	48.33 (2.94)	51.33 (3.33)	0.33 (0.52)	51.67 (2.94)	51.50	**0.0107 (13.13)**
	0.4	31.17 (3.76)	67.17 (3.66)	1.67 (1.37)	68.83 (3.76)	**69.00** ^**a**^	
	2	33.17 (4.07)	57.50 (10.62)	9.33 (7.94)	66.83 (4.07)	65.50	
	10	30.17 (13.21)	61.00 (10.18)	8.83 (4.36)	69.83 (13.21)	**75.00** ^**a**^	
	50	33.67 (4.50)	63.17 (4.71)	3.17 (3.71)	66.33 (4.50)	67.50	
Etoposide	50	3.50 (1.42)	6.83 (4.71)	89.67 (5.20)	96.50 (1.52)	**96.50** ^**a**^	

Paraoxon	0	78.00 (10.12)	21.00 (8.92)	1.00 (1.67)	22.00 (10.12)	21.00	**0.0054 (14.68)**
	0.2	57.33 (17.28)	40.67 (15.93)	2.00 (1.79)	42.67 (17.28)	41.00	
	1	43.33 (13.31)	52.33 (12.23)	4.33 (3.67)	56.67 (13.31)	**56.00** ^**a**^	
	5	52.00 (5.22)	43.33 (5.47)	4.67 (3.72)	48.00 (5.22)	48.00	
	25	42.33 (15.92)	43.67 (24.57)	14.00 (12.71)	57.67 (15.92)	**60.00** ^**a**^	
Etoposide	50	7.00 (4.86)	4.33 (2.34)	88.67 (5.32)	93.00 (4.86)	**91.00** ^**b**^	

Permethrin	0	55.33 (6.15)	42.67 (6.15)	2.00 (0.00)	44.67 (6.15)	47.00	**0.0092 (13.46)**
	8	29.00 (16.19)	65.67 (13.65)	5.33 (5.75)	71.00 (16.19)	**73.00** ^**a**^	
	40	36.00 (6.33)	61.00 (5.90)	3.00 (2.10)	64.00 (6.33)	64.00	
	200	24.33 (11.13)	70.33 (7.31)	5.33 (6.02)	75.67 (11.13)	**76.00** ^**a**^	
	1000	36.33 (18.82)	60.00 (16.44)	3.67 (2.94)	63.67 (18.82)	64.00	
Etoposide	50	0.33 (0.82)	7.00 (3.29)	92.67 (3.01)	99.67 (0.82)	**100.00** ^**b**^	

Endosulfan lactone	0	55.33 (6.15)	42.67 (6.15)	2.00 (0.00)	44.67 (6.15)	47.00	**0.0001 (23.30)**
	0.08	32.67 (5.61)	62.33 (7.31)	5.00 (4.52)	67.33 (5.61)	66.00	
	0.4	27.00 (5.33)	70.33 (4.46)	2.67 (2.42)	73.00 (5.33)	**74.00** ^**a**^	
	2	27.00 (5.33)	73.67 (3.88)	3.33 (2.73)	77.00 (3.03)	**77.00** ^**a**^	
	10	47.00 (8.08)	51.67 (4.53)	1.33 (1.63)	53.00 (8.07)	53.00	
Etoposide	50	0.33 (0.82)	7.00 (3.27)	92.67 (3.01)	99.67 (0.82)	**100.00** ^**b**^	

PCP	0	78.00 (10.12)	21.00 (8.92)	1.00 (1.67)	22.00 (10.12)	21.00	**0.0259 (11.06)**
	0.03	48.33 (14.11)	49.00 (13.19)	2.67 (1.63)	51.67 (14.11)	52.00	
	0.15	39.67 (16.71)	56.33 (17.45)	4.00 (2.83)	60.33 (16.71)	**60.00** ^**a**^	
	0.75	62.67 (23.31)	34.00 (22.52)	3.33 (2.73)	37.33 (23.31)	30.00	
	3.75	68.67 (23.45)	29.00 (23.07)	2.33 (2.34)	31.33 (23.45)	31.00	
Etoposide	50	7.00 (4.86)	4.33 (2.34)	88.67 (5.32)	93.00 (4.86)	**91.00** ^**b**^	

^*∗*^Kruskal-Wallis test considering all damaged cells; d.f. 4 in all tests;  ^a^significant dose compared with the negative control; post hoc Dunn's test, *p* < 0.05.  ^b^Etoposide results were always significant: Mann–Whitney test, *p* < 0.05. PCP: pentachlorophenol; only compounds with a positive result are presented here considering a *p* value of <0.05 for significance. See text.

**Table 3:** Cytotoxic effect of pesticides and metabolites that induced double-strand breaks.

	Compound	Survival range (%)	*R* ^2^	*F*	*p* value^*∗*^
Pesticide	Glyphosate	**100 to 70**	**0.252**	**9.431**	**0.0047**
	Permethrin	100 to 100	0.006386	0.06427	0.8050
	Pentachlorophenol	100 to 78	0.007	0.092	0.7669

Metabolite	Endosulfan lactone	**100 to 60**	**0.6787**	**25.35**	**0.0003**
	Paraoxon	98 to 90	0.1473	2.246	0.1579

Positive control	Etoposide	100 to 96	5.388*e* − 006	7.004*e* − 005	0.9934

^*∗*^Linear regression; the positive control was not cytotoxic at the concentration tested, 50 *µ*M.

**Table 4:** Detection of Ku80 phosphorylated and Rad51 proteins in mononuclear cells treated with pesticides and metabolites that induced *γ*-H2AX foci.

Compound	Dose (*µ*M)	P-Ku80			Rad51		
		Mean OD (SD)	Median OD	*p* ^*∗*^ * (chi-squared value)*	Mean OD (SD)	Median OD	*p* ^*∗*^ * (chi-squared value)*
Glyphosate	0	100.00 (34.48)	104.05	**0.0297 (7.985)**	100.00 (47.93)	104.17	0.4955 (2.559)
	1.25	200.0 (63.99)	206.20		134.4 (34.57)	122.89	
	2.5	227.3 (98.69)	190.91		140.8 (41.92)	158.29	
	5	253.9 (130.40)	**205.2**7^**a**^		107.5 (49.28)	113.41	
	Etoposide^*∗∗*^	214.2 (48.55)	**198.21**	**0.028**6^**b**^	119.3 (11.74)	84.26	0.6571

Paraoxon	0	100.0 (22.11)	102.55	**0.0077 (9.551)**	100.0 (50.54)	96.74	0.3812 (3.243)
	1	186.64 (39.72)	**196.4**3^**a**^		178.8 (73.36)	171.4	
	5	155.32 (17.06)	**159.1**3^**a**^		115.9 (6.84)	113.0	
	25	154.58 (8.76)	**157.7**9^**a**^		180.6 (95.02)	147.7	
	Etoposide^*∗∗*^	155.4 (63.53)	167.30	0.1044	109.5 (25.86)	98.03	0.6571

^*∗*^Kruskal-Wallis test: P-Ku80 was significant in glyphosate and paraoxon; *p* < 0.05; d.f. 3 in all tests;  ^a^significant dose compared with the negative control; post hoc Dunn's test: *p* < 0.05;  ^*∗∗*^Mann–Whitney test: ^b^*p* < 0.05; OD: optical density. The results presented correspond to two separate experiments; only compounds who were positive are presented. See text.
